# Emergency Survey Methods in Acute Cryptosporidiosis Outbreak

**DOI:** 10.3201/eid1105.040871

**Published:** 2005-05

**Authors:** LeAnne M. Fox, M. Cheryl Banez Ocfemia, D. Charles Hunt, Brian G. Blackburn, Daniel Neises, W. Kay Kent, Michael J. Beach, Gianfranco Pezzino

**Affiliations:** * Centers for Disease Control and Prevention, Atlanta, Georgia, USA;; † Kansas Department of Health and Environment, Topeka, Kansas, USA;; ‡ University of Kansas Medical Center, Kansas City, Kansas, USA;; § Lawrence-Douglas County Health Department, Lawrence, Kansas, USA;; ¶ Kansas Health Institute, Topeka, Kansas, USA

**Keywords:** cryptosporidiosis, case-control, telephone survey, recreational water, computer-assisted telephone interview

## Abstract

In August 2003, a communitywide outbreak of cryptosporidiosis occurred in Kansas. We conducted a case-control study to assess risk factors associated with *Cryptosporidium* infection by using the telephone survey infrastructure of the Behavioral Risk Factor Surveillance System. Using existing state-based infrastructure provides an innovative means for investigating acute outbreaks.

Internet-based computerized questionnaire administration has been increasingly used in epidemiologic investigations and can reduce the resources and workload required for these studies (1,2). Computer-assisted telephone interview (CATI) surveys have been used for a variety of population and national health surveys, but their use in acute infectious disease outbreak investigations has not been reported. These surveys have the advantage of computerized data entry and trained interviewers, which minimizes data entry errors and interview time. These surveys also provide a readily accessible computerized database for data analysis. CATI systems facilitate telephone survey administration, provide sophisticated record management, and allow close monitoring of study progress.

*Cryptosporidium* spp. are chlorine-resistant, protozoan parasites that cause prolonged watery diarrhea. Outbreaks of cryptosporidiosis have historically been associated with recreational water (3–7), day camps (8), and daycare facilities (9–11). In August 2003, a communitywide cryptosporidiosis outbreak occurred in Douglas County, Kansas, USA. We conducted a community-based epidemiologic investigation to determine the risk factors associated with *Cryptosporidium* infection by using the infrastructure of the Health Risk Studies Program at the Kansas Department of Health and Environment (KDHE). This program conducts the Behavioral Risk Factor Surveillance System (BRFSS) and other telephone surveys within the state health department rather than through a contract with an external research organization. We assessed the feasibility of using a CATI system to conduct a case-control study in an acute outbreak investigation and identified areas to improve the use of this system.

## The Study

In late August 2003, local, state, and federal health officials began an investigation to determine the risk factors associated with an outbreak of cryptosporidiosis and to develop interventions to control it. The epidemiologic investigation resulted in 96 laboratory-confirmed cases of *Cryptosporidium* infection and >600 clinical cryptosporidiosis cases.

BRFSS is an established nationwide population-based telephone survey system that primarily measures behavioral risk factors associated with leading causes of death. It is currently the largest continuous telephone survey in the world; it expanded to all 50 states in 1993 (http://www.cdc.gov/brfss). In Kansas, the Health Risk Studies Program conducts the BRFSS in-house and provides the capacity and expertise to design and implement special surveys. During this outbreak investigation, KDHE, the Lawrence-Douglas County Health Department, and the Centers for Disease Control and Prevention (CDC) used the BRFSS infrastructure, which consisted of a fully networked, computer-assisted telephone interviewing system (WinCATI Sawtooth Technologies, Northbrook, IL, USA) to conduct a case-control study.

We conducted a matched case-control study to identify specific risk factors for infection. Laboratory-confirmed case-patients were identified through laboratory surveillance. Clinical cryptosporidiosis patients were identified during the case ascertainment portion of the study, by surveying households of elementary school children and persons who had sought healthcare for diarrheal symptoms. All laboratory-confirmed patients were enrolled, as were a random selection of clinical cryptosporidiosis patients within 4 age strata. Two controls were matched to each patient, and each control was asked the same questions for the specific exposure period of the patient to whom they were matched. A maximum of 1 case-patient or control-patient per household was enrolled.

**Figure 1:**
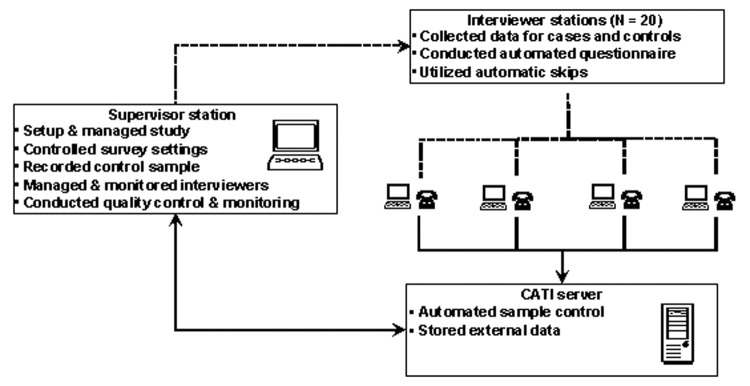
Kansas Health Risk Studies Program computer-assisted telephone interview (CATI) system architecture for case-control study.

The CATI system relies on a networked central server with both interviewer and supervisory stations (Figure 1). The system allows for questionnaire programming, record management, scheduling of calls, and monitoring the disposition of calls. Telephone numbers for patients were programmed into the CATI system; controls were identified by random digit dialing. Telephone numbers, which included all telephone exchanges represented in the 100,000-person county, were purchased from a commercial survey research sample provider. This sample was prescreened to remove both business and nonworking numbers but did include unlisted numbers. All residential numbers in the community were eligible, and the CATI system released the telephone numbers randomly. Frequency matching controls to patients was performed by inquiring for a person at the residence within a certain age range. The questionnaire was programmed into the CATI system by using both range and logic checks, to minimize data entry errors, as well as skip patterns, which allow the respondent to answer only those questions that pertain to them. Twenty BRFSS personnel were trained in two 1-hour training sessions on the use of the outbreak instrument and conducted pilot testing of the questionnaire for appropriate wording and skip patterns.

This case-control study was initiated within 8 days of finalizing the questionnaire. Approximately 11,400 telephone calls were made, and 770 interviewer hours were used in a 41-day period to complete 151 case-patient and 302 control interviews. The average interview length for completion of the case questionnaire was 28 minutes, and the average interview length for completion of the control questionnaire was 16 minutes (Table). Data from the investigation showed multiple risk factors associated with *Cryptosporidium* infection, including exposure to several recreational water venues.

**Table:** Utilization of CATI system for case-control study*

Characteristic	Case-patients	Controls
Interview period	Sep 15–Sep 29	Sep 28–Oct 21
No. enrolled	151	302
No. calls made	1,357	10,101
No. refusals	56	330
Average interview length (min)	28	16
Interviewer hours	263	508

*CATI, computer-assisted telephone interview.

## Conclusions

This study highlights the feasibility and potential benefits of a coordinated effort between chronic and infectious disease sections at local, state, and federal public health agencies in responding to an acute infectious disease outbreak. We used existing infrastructure and resources in the chronic disease division of a state health department to conduct a communitywide case-control study. To our knowledge, this is the first time a CATI system based at a state health department has been used to respond to an acute infectious disease outbreak. The BRFSS program at KDHE has facilitated the development of the internal expertise and infrastructure necessary to design and implement large-scale and complex telephone surveys. This program includes providing a cohort of trained interviewers who could efficiently collect data to allow a comprehensive assessment of the risk factors associated with *Cryptosporidium* infection in this outbreak.

With the WinCATI system, interviewers were able to enter questionnaire data directly into the computerized system in real time, thus creating a database that could be easily converted into a variety of statistical programs for data analysis. This system obviated the need for paper questionnaires and subsequent data entry. The questionnaire was programmed to require certain data before proceeding (logic checks) or to warn the user of an incorrect entry (data checks), thus decreasing the possibility of missing or including incorrect data. Use of an existing infrastructure did not require immediate recruitment and training of volunteer interviewers, the traditional method for outbreak investigations, but provided a trained interviewing staff. Additionally, this mechanism liberated the professional public health staff to focus their efforts on the multifaceted public health interventions required in a communitywide outbreak.

The use of existing CATI systems may be of value in several circumstances. As demonstrated here, in large, communitywide outbreaks, CATI systems can provide substantial resources and personnel capacity that may substantially enhance investigation efforts in responding to a public health threat. Additionally, CATI systems, similar to BRFSS, are well-suited for performing long-term studies, for on-going studies attempting to determine the source of sporadic infectious disease cases, and for public health surveillance. They can also provide a practical means of obtaining controls for case-control studies.

Nevertheless, several limitations should be noted about the use of population-based telephone surveys in responding to acute outbreak scenarios. Unlike traditional communicable disease control programs, community telephone survey efforts, such as BRFSS, were not created for immediate response, and therefore their use in this context has some limitations. These include the time required to program the questionnaire into a CATI system and the organization of professional staff time in an outbreak situation. The preprogramming of generic infectious disease outbreak questionnaire modules (e.g., demographics, clinical symptoms, or foodborne or waterborne exposures) into a CATI system may help decrease the start-up time required for questionnaire implementation.

CATI surveys may also be less useful in several circumstances. These include smaller focal outbreaks in which the use of many resources and lengthy start-up times would be disadvantageous; particularly when these investigations are within the capacity of existing communicable disease programs. CATI surveys also have the standard limitations and biases inherent in telephone surveys. These include the following: selection bias, inclusion of only those who have a home telephone number; and response bias. In addition, those who participate may be different from those unwilling to participate, and declining response rates have been noted among telephone surveys (12). Moreover, the regular use of a CATI infrastructure, like BRFSS, for acute outbreak situations needs to be further assessed to prevent it from detracting from standard BRFSS activities. CATI systems, therefore, may not replace existing disease investigation programs but have the potential to supplement these programs.

Using existing state-based infrastructure in the chronic disease arena should be considered as a potential response strategy for future public health emergencies, and state health departments should consider developing plans and identifying financial resources for implementing similar strategies when performing large-scale investigations. Because many state health departments may contract with a survey research firm to perform population-based telephone surveys, including reference to special studies related to urgent public health needs should be included in these contract negotiations. Using CATI systems provides an innovative and potentially valuable adjunct to current outbreak investigation methods and should be considered as a viable addition or alternative for conducting acute outbreak investigations, particularly during large-scale, emergency situations when resources are limited.
